# Co-Administration of Clomiphene Citrate and Letrozole in Mild Ovarian Stimulation Versus Conventional Controlled Ovarian Stimulation Among POSEIDON Group 4 Patients

**DOI:** 10.3389/fendo.2021.780392

**Published:** 2022-01-14

**Authors:** Hsin-Ta Lin, Meng-Hsing Wu, Li-Chung Tsai, Ta-Sheng Chen, Huang-Tz Ou

**Affiliations:** ^1^ Department of Obstetrics and Gynecology, National Cheng Kung University Hospital, College of Medicine, National Cheng Kung University, Tainan, Taiwan; ^2^ Department of Obstetrics and Gynecology, College of Medicine, National Cheng Kung University, Tainan, Taiwan; ^3^ Institute of Clinical Pharmacy and Pharmaceutical Sciences, College of Medicine, National Cheng Kung University, Tainan, Taiwan; ^4^ School of Pharmacy, College of Medicine, National Cheng Kung University, Tainan, Taiwan

**Keywords:** clomiphene citrate, letrozole, mild stimulation, POSEIDON Group 4, *in vitro* fertilization

## Abstract

This retrospective study assessed the effect of the co-administration of clomiphene citrate (CC) and letrozole in mild ovarian stimulation, compared to conventional regimens, among Patient-Oriented Strategies Encompassing Individualized Oocyte Number (POSEIDON) Group 4 patients. There were 114 POSEIDON Group 4 patients undergoing *in vitro* fertilization treatments with 216 stimulation cycles recruited from a Taiwan’s reproductive center during 2016-2020. Main outcomes were the numbers, quality of retrieved oocytes and embryo development. Pregnancy outcomes were assessed after embryo transfers. Per stimulation cycle, patients receiving mild stimulation with a combination of CC and letrozole (study group) versus those with COS (control group) had lower numbers of pre-ovulatory follicles (2.00 ± 1.23 vs. 2.37 ± 1.23, *p*=0.0066) and oocytes retrieved (1.83 ± 1.17 vs. 2.37 ± 1.23, *p*=0.0017), and lower follicular output rate (58.6% vs. 68.38%, *p*=0.0093) and mature oocyte output rate (44.29% vs. 52.88%, *p*=0.0386) but a higher top-quality metaphase II oocyte ratio (66.7% vs. 54.59%, *p*=0.0444) and a similar fertilization rate (91.67% vs. 89.04%, *p*=0.4660). With adjustment for significant between-group baseline differences using multivariable logistic generalized estimating equation model analyses, there was no statistical difference in oocytes retrieved and embryo development between the study and control groups, and insignificant increases in successful pregnancies in the study group were found compared to the control group (i.e., odds ratios [95% CIs]: 1.13 [0.55, 232] and 1.50 [0.65, 3.49] for ongoing pregnancy and live birth, respectively). For POSEIDON Group 4 patients, cotreatment of CC and letrozole in mild stimulation may increase the high-quality oocyte ratio and yield comparable fertilization rate and pregnancy outcomes.

## Introduction

According to the Patient-Oriented Strategies Encompassing Individualized Oocyte Number (POSEIDON) criteria, approximately 47% of patients have low prognosis in assisted reproductive technology treatment (1). The POSEIDON classified poor ovarian response (POR) patients into four subgroups and the Group 4 patients (i.e., patients aged≥35 years, antral follicle count [AFC]<5, and anti-M̈llerian hormone [AMH]<1.2 ng/ml) account for 55% of low prognosis patients (1) and have the poorest ovarian responses (e.g., low number of oocytes retrieved, limited number of embryos produced, and no high-quality embryos for transfer). The POSEIDON provides detailed classification for POR patients, which reduces the heterogeneity seen in the Bologna criteria (2), and clinical recommendations to promote individualized treatment.

Treating POSEIDON Group 4 patients is challenging for clinicians, and current strategies mainly rely on evidence from general POR patients (3). Various ovarian stimulation protocols have been employed to enhance the number and quality of oocytes as well as pregnancy rates. Mild ovarian stimulation for *in vitro* fertilization (IVF) combines oral agents such as clomiphene citrate (CC) or aromatase inhibitors (e.g., letrozole) with relatively low-dose exogenous gonadotropins (4). CC is a nonsteroidal selective estrogen receptor modulator with both estrogen agonist and antagonist properties, and letrozole is a potent, competitive, nonsteroidal aromatase inhibitor.

Previous studies showed that mild stimulation with CC and lower doses of gonadotropins among POR patients was comparable or not inferior to conventional ovarian stimulation (COS) with higher doses of gonadotropins in terms of pregnancy outcomes (5, 6). Mild stimulation with letrozole and lower doses of gonadotropins yielded similar pregnancy rates and numbers of oocytes retrieved and embryos transferred compared to those obtained with COS with higher doses of gonadotropins (7, 8). The rationale for using CC or letrozole during IVF treatment is to provide gentle stimulation with relatively low-dose gonadotropin use to decrease the cost of IVF and the time required for stimulation to minimize patient discomfort.

However, the number of study patients in previous studies (5–8) was generally small, which decreases the statistical power to detect the significance of study outcomes (especially pregnancy outcomes). No studies have focused on POSEIDON Group 4 and none have assessed the clinical effects of the cotreatment of CC and letrozole in mild stimulation versus COS. CC and letrozole both stimulate gonadotropin-releasing hormone (GnRH) secretion by decreasing the negative feedback effect of estrogen. CC depletes central estrogen receptors whereas letrozole decreases estrogen production directly and increases androgen level in the ovary (9). These different mechanisms in mild stimulation may yield a synergistic effect to improve the IVF outcomes of POR patients.

Against this background, we assessed the clinical effects of the co-administration of CC and letrozole in mild stimulation versus COS in terms of the number and quality of oocytes retrieved and embryo development as well as subsequent pregnancy outcomes.

## Material and Methods

Before commencement of the study, permission was obtained from the Institutional Review Board (IRB) of National Cheng Kung University Hospital (NCKUH), Tainan, Taiwan (A-ER-109-431). The infertility treatment pertaining to this study was carried out in accordance with relevant guidelines and regulations. The informed consents to study patients were waived by the IRB of NCKUH considering that all study procedures were routine care and practice.

### Study Patients

**Figure 1:**
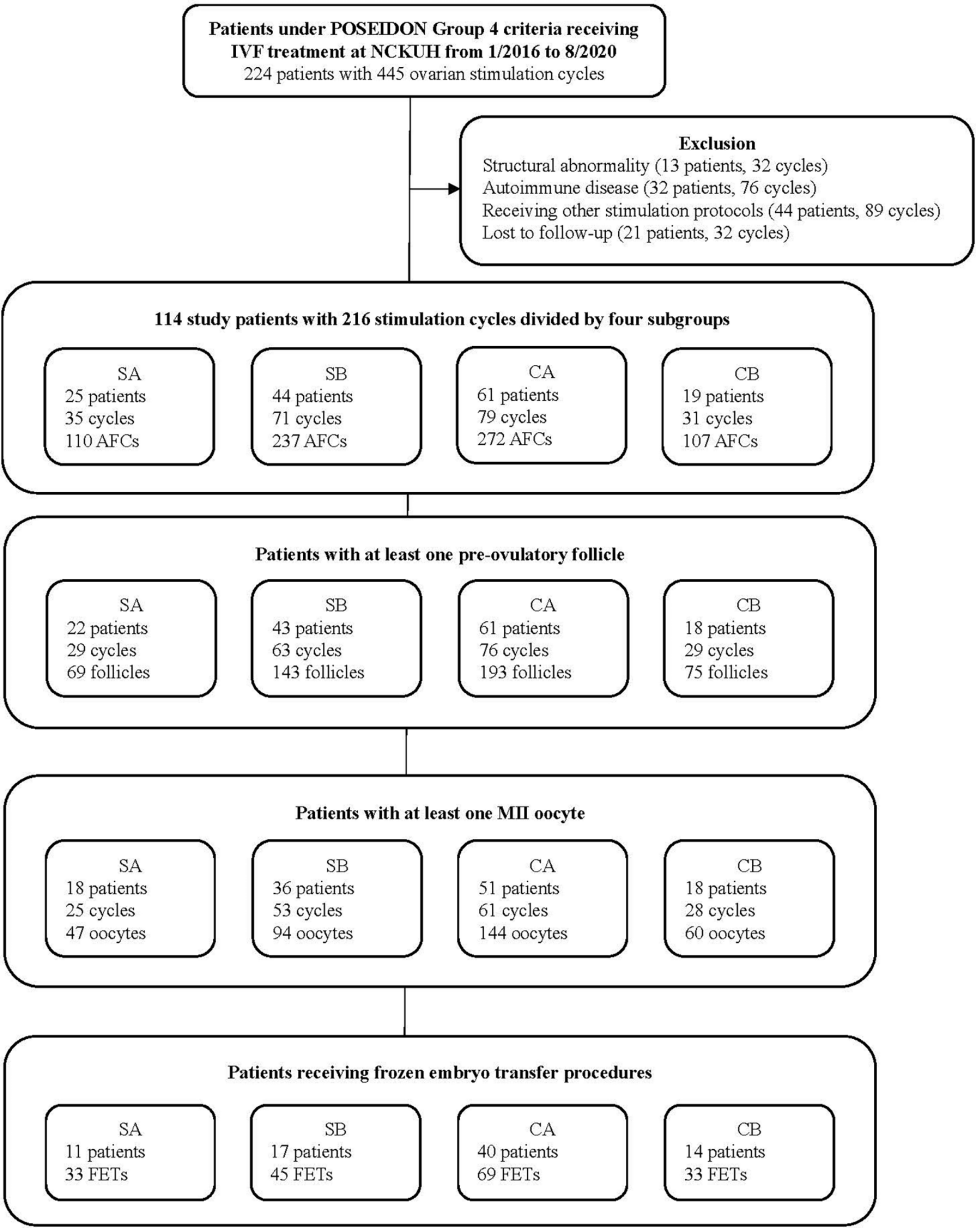
Patient selection flow chart. POSEIDON, Patient-Oriented Strategies Encompassing IndividualizeD Oocyte Number; IVF, *in vitro* fertilization; NCKUH, National Cheng Kung University Hospital; SA, study group receiving administration of clomiphene citrate and letrozole in mild ovarian stimulation with gonadotropin-releasing hormone (GnRH) antagonist protocol; SB, study group receiving administration of clomiphene citrate and letrozole in mild ovarian stimulation with progestin-primed ovarian stimulation (PPOS) protocol; CA, control group receiving conventional ovarian stimulation with the GnRH antagonist protocol; CB, control group receiving conventional ovarian stimulation with the PPOS protocol; AFC, antral follicle count; MII, metaphase II; FET, frozen embryo transfer.

This retrospective cohort study included patients who 1) were undergoing IVF/intracytoplasmic sperm injection cycles between January 2016 and August 2020 at NCKUH, 2) met the POSEIDON Group 4 criteria (10) (i.e., aged ≥35 years, total AFC <5, and AMH <1.2 ng/ml), 3) were receiving mild stimulation with a lower dose of gonadotropin (150~225 IU/daily) and combination of CC and letrozole or COS with a higher dose of gonadotropin (300~450 IU/daily), and 4) had endometrial thickness at basal evaluation (Day 2-3) less than 6 mm. Patients with the following characteristics were excluded: 1) structural abnormalities (e.g., uterine malformation, intrauterine adhesion, severe adenomyosis, or grade 3 or higher endometriosis), 2) concomitant diseases including hypercholesterolemia, insulin resistance and hypertension, or smoking habits, which may influence the ovarian stimulation outcomes, 3) autoimmune-related recurrent pregnancy loss or recurrent implantation failure, 4) receiving other types of ovarian stimulation, or 5) lost to follow-up. Patients were classified into two groups: the study group, which received mild stimulation with a combination of CC and letrozole, and the control group, which received COS without CC or letrozole. Two protocols, GnRH antagonists and progestin-primed ovarian stimulation (PPOS), are commonly used in ovarian stimulation to prevent premature LH surge, and they may yield differential effects on IVF treatment outcomes (11–14). With this regard, study patients were further divided into four subgroups: 1) study group with protocol A (SA) (patients receiving GnRH antagonists in mild stimulation), 2) study group with protocol B (SB) (patients receiving PPOS in mild stimulation), 3) control group with protocol A (CA) (patients receiving conventional GnRH antagonist protocol), and 4) control group with protocol B (CB) (patients receiving conventional PPOS protocol) (**Figure 1**).

### Stimulation Protocols and Embryo Transfer Procedures

The four ovarian stimulation protocols (i.e., SA, SB, CA, and CB) are detailed in **Supplementary Figure 1** (including dosages, and day and route of drug administration). Briefly, CC and letrozole were taken by the study group from day 3 of the menstruation cycle. The daily dose of gonadotropins was adjusted for individual patients. Dydrogesterone (in the PPOS protocol) was used daily from day 3 of the menstruation cycle or cetrorelix (Cetrotide^®^, Merk Serono, Darmstadt, Germany) (in the GnRH antagonist protocol) was administered daily when the dominant follicle reached 13 mm in size until the day of human chorionic gonadotropin (hCG) administration. Frozen embryo transfer (FET) was performed when at least two high-quality embryos were obtained after ovarian stimulation. Only two embryos were transferred per cycle.

### Study Outcomes and Measures

The primary study outcome was ovarian stimulation outcomes in terms of the quality of an individual follicle or oocyte, measured by the following indicators: 1) being a pre-ovulatory follicle, 2) being a metaphase II (MII) oocyte, 3) being a top-quality MII oocyte (i.e., appearance the oocyte as a clear, moderate granular cytoplasm, a clear/colorless zona pellucida, a small perivitelline space and containing a single unfragmented polar body (15) and 4) being an embryo with two-pronuclear zygotes (2PN). Several measures per stimulation cycle were also estimated: follicular output rate (FORT) (i.e., number of pre-ovulatory follicles/AFC × 100%), mature oocyte output rate (MOOR) (i.e., number of MII oocytes/AFC × 100%), top-quality MII oocyte ratio (i.e., number of top-quality MII oocytes/total number of MII oocytes × 100%), and fertilization rate (i.e., number of embryos with 2PN)/number of MII oocytes). Pregnancy results following the FET cycle were the secondary outcomes: 1) ongoing pregnancy, which was ascertained by the appearance of a gestational sac with a viable fetal heartbeat at the 10^th^ week of gestational age, and 2) live birth, which was defined as the presence of a live fetus (or feti) after the 24^th^ week of gestational age.

Patient baseline characteristics were also measured at the time of beginning IVF treatment (i.e., stimulation cycle) including maternal age, body mass index (BMI), anti-Müllerian hormone (AMH), basal E2 and FSH, the length of ovarian stimulation, endometrial thickness and E2 level at day of hCG administration, infertility year, the grade of transferred embryo, and embryo transfer failure history (**Tables 1** and **2**). Moreover, the cancellation of ovarian stimulation for a patient was confirmed when the patient had any following status: 1) failure to ovarian stimulation under gonadotropin or CC and letrozole stimulation, which was defined as no follicle larger than 10 mm till the late follicular phase, 2) premature LH surge, 3) intolerance to or having adverse effects about ovarian stimulation medications and then discontinuation of the treatment. The cancellation rate for each study group was measured and compared.

**Table 1:** Patient characteristics stratified by treatment protocol among patients receiving stimulation protocols.

Characteristics	Study group (55 patients, 106 cycles)		Control group (73 patients, 110 cycles)		Difference	Protocol A (79 patients, 114 cycles)		Protocol B (59 patients, 102 cycles)		Difference
	N	Mean (sd) or %	N	Mean (sd) or %	*p*-value	N	Mean (sd) or %	N	Mean (sd) or %	*p*-value
Maternal age at IVF treatment (years)	106	42.62 (3.03)	110	40.72 (3.30)	<.0001^***^	114	41.37 (3.29)	102	41.97 (3.31)	0.1822
BMI (kg/m^2^)	106	23.03 (3.37)	110	22.95 (3.54)	0.8614	114	23.61 (3.81)	102	22.31 (2.87)	0.0049^**^
AMH (ng/mL)	103	0.38 (0.30)	110	0.58 (0.32)	<.0001^***^	112	0.52 (0.32)	101	0.44 (0.32)	0.0767
Basal E2 (pg/mL)	105	73.16 (114.61)	109	70.17 (126.32)	0.8561	112	49.25 (90.06)	102	96.22 (143.22)	0.0051^**^
Basal FSH (pg/mL)	106	13.43 (8.68)	110	10.26 (4.49)	0.0010^**^	114	12.12 (6.14)	102	11.48 (7.93)	0.5078
Length of ovarian stimulations (days)	106	10.27 (2.98)	110	9.00 (2.19)	0.0005^***^	114	9.25 (2.52)	102	10.03 (2.80)	0.0339^*^
Endometrial thickness at day of hCG administration (mm)	78	8.64 (1.58)	102	9.87 (2.05)	<0.0001^***^	102	9.49 (1.86)	78	9.14 (2.07)	0.2402
E2 level at day of hCG administration (pg/mL)	93	250.85 (257.81)	108	645.49 (392.35)	<.0001^***^	109	510.37 (401.71)	92	406.64 (369.20)	0.0581
Total dosage of gonadotropins in stimulation without corifollitropin alfa (Elonva^®^) (IU)	106	688.92 (473.77)	35	1555.71 (455.97)	<.0001^***^	47	852.13 (624.45)	94	930.05 (589.13)	0.4786
Total dosage of gonadotropins in stimulation with Elonva® (IU)	–	–	75	601.00 (447.37)	–	67	592.16 (455.30)	8	675.00 (392.79)	0.5927
Cancellation rate (%)	105	12.38 (13/105)	107	5.61 (6/107)	<.0001^***^	112	8.93 (10/112)	100	9.00 (9/100)	0.9855
Stimulation outcomes per cycle										
AFC	106	3.27 (0.92)	110	3.45 (0.89)	0.7604	114	3.35 (1.00)	102	3.37 (0.81)	0.8601
No. of pre-ovulatory follicles	106	2.00 (1.23)	110	2.44 (1.10)	0.0066**	114	2.30 (1.17)	102	2.14 (1.19)	0.3200
No. of OPU	94	1.83 (1.17)	105	2.37 (1.23)	0.0017^**^	106	2.15 (1.29)	93	2.08 (1.15)	0.6631
No. of MII oocytes	92	1.53 (1.07)	104	1.96 (1.22)	0.2074	104	1.84 (1.24)	92	1.67 (1.09)	0.3297
No. of 2PN embryos	87	1.48 (1.06)	91	1.95 (0.94)	0.0023^**^	90	1.82 (1.02)	88	1.61 (1.01)	0.1729
FORT (%)	104	58.65	107	68.38	0.0093^**^	109	66.82	102	60.13	0.0749
MOOR (%)	92	44.29	104	52.88	0.0386^*^	104	50.64	92	46.83	0.3586
Top-quality MII oocyte ratio (%)	78	66.67	89	54.59	0.0444^*^	86	54.46	81	66.36	0.0452^*^
Fertilization rate (%)	78	91.67	89	89.04	0.4660	86	87.31	81	93.42	0.0822

sd, standard deviation; IVF, in vitro fertilization; BMI, body mass index; AMH, anti-Müllerian hormone; FSH, follicle-stimulating hormone; hCG, human chorionic gonadotropin; AFC, antral follicle count per stimulation cycle; OPU, ovarian pick-up; MII, metaphase II; 2PN, two-pronuclear zygote; FORT, follicular output rate; MOOR, mature oocyte output rate. Study group receiving cotreatment of clomiphene citrate and letrozole in mild ovarian stimulation. Control group receiving conventional ovarian stimulation, Protocol A, where patients received ovarian stimulation with gonadotropin-releasing hormone (GnRH) antagonist protocol, Protocol B, where patients received ovarian stimulation with progestin-primed ovarian stimulation (PPOS) protocol.

•“Cycle” refers to a stimulation cycle and “N” refers to the number of stimulation cycles.

•FORT (follicular output rate) was defined as the number of pre-ovulatory follicles (a follicle with size ≥18 mm) divided by the total number of follicles per cycle.

•MOOR (mature oocyte output rate) was defined as the number of mature oocytes divided by the total number of follicles per cycle.

•Top-quality MII oocyte ratio was defined as the number of top-quality oocytes divided by the total number of oocytes per cycle.

•Fertilization rate was measured as the number of oocytes developing into two-pronuclear zygote embryos divided by the total number of oocytes per cycle.

•Difference in patient characteristics was tested using *t*-tests (for continuous variables) and chi-square tests (for dichotomous and categorical variables).

•^*^ indicates *p*-value <0.05, ^**^ indicates *p*-value <0.01, and ^***^ indicates *p*-value <0.001.

**Table 2:** Patient characteristics stratified by treatment protocol among patients receiving embryo transfer cycles.

Characteristics	Study group (28 patients, 78 FET cycles)		Control group (54 patients, 102 FET cycles)		Difference study vs. control	Protocol A (48 patients, 102 FET cycles)		Protocol B (30 patients, 78 FET cycles)		Difference protocol A vs. B
	N	Mean (sd) or %	N	Mean (sd) or %	*p*-value	N	Mean (sd) or %	N	Mean (sd) or %	*p*-value
Maternal age at FET (years)	78	42.71 (3.36)	102	40.66 (3.11)	<0.0001^***^	102	41.12 (3.32)	78	42.1 (3.38)	0.0523
BMI (kg/m^2^)	78	22.17 (2.51)	102	22.88 (3.43)	0.1062	102	23.52 (3.46)	78	21.34 (1.89)	<0.0001^***^
Infertility year (year)	78	5.22 (2.94)	102	5.14 (2.95)	0.8579	102	5.17 (2.88)	78	5.17 (3.02)	0.9996
AMH (ng/mL)	78	0.35 (0.29)	102	0.54 (0.31)	<0.0001^***^	102	0.48 (0.33)	78	0.44 (0.29)	0.4460
No. 1 transferred embryo grade	78	1.33 (0.47)	102	1.33 (0.47)	1.0000	102	1.31 (0.47)	78	1.36 (0.48)	0.5280
No. 2 transferred embryo grade	78	1.91 (0.46)	102	1.96 (0.53)	0.4938	102	1.97 (0.5)	78	1.9 (0.50)	0.3302
Sum of transferred embryo grades	78	3.22 (0.73)	102	3.29 (0.78)	0.5019	102	3.27 (0.75)	78	3.24 (0.78)	0.7881
ET failure history										
None	20	25.64%	30	29.41%	0.5757	30	29.41%	20	25.64%	0.5757
At least one	58	74.36%	72	70.59%		72	70.59%	58	74.36%	
Pregnancy outcomes										
Ongoing pregnancy rate (%)	19	24.36% (19/78)	22	21.57% (22/102)	0.6583	21	20.59% (21/102)	20	25.64% (20/78)	0.4231
Live birth rate (%)	15	19.23% (15/78)	14	13.73% (14/102)	0.3195	14	13.73% (14/102)	15	19.23% (15/78)	0.3195

sd, standard deviation; FET, frozen embryo transfer; BMI, body mass index; AMH, anti-Müllerian hormone; S, study group receiving cotreatment of clomiphene citrate and letrozole in mild ovarian stimulation; C, control group receiving conventional ovarian stimulation; A, protocol A, where patients received ovarian stimulation with gonadotropin-releasing hormone (GnRH) antagonist protocol; B, protocol B, where patients received ovarian stimulation with progestin-primed ovarian stimulation (PPOS) protocol.

•Definition of embryo grade definition: 1 = an embryo with excellent quality and no fragmentation; 2 = an embryo with good quality and 1%-20% fragmentation; and 3 = an embryo with fair quality and 21%-50% fragmentation.

•Difference in patient characteristics was tested using *t*-tests (for continuous variables) and chi-square tests (for dichotomous and categorical variables).

•^***^ indicates *p*-value <0.001.

### Statistical Analyses

Associations between different stimulation protocols (i.e., study group versus control group) and individual study outcomes (e.g., obtaining a 2PN embryo, achieving a successful ongoing pregnancy) were evaluated using multivariable logistic generalized estimating equation (GEE) model analyses with adjustment for potential confounding factors for study outcomes. Given repeated nature of study data (i.e., stimulation cycles, ET cycles) within a person, the GEE analysis was appropriate (16–18). Associations between all individual patient baseline characteristics and study outcomes were first examined through univariable GEE model analyses. Characteristics with significant associations were then adjusted in multivariable GEE models for associations between stimulation protocols (e.g., study group versus control group) and study outcomes. The statistical results of GEE model analyses are presented as odds ratios (ORs) and 95% confidence intervals (CIs). A two-tailed *p*-value of <0.05 was considered to indicate a statistically significant difference. All analyses were carried out using SAS software version 9.4.

## Results

### Patient Selection

A total of 114 cases (with 216 ovarian stimulation cycles) were included in analysis. 25, 44, 61, and 19 patients were classified into the study group with GnRH antagonist (SA), study group with PPOS (SB), control group with GnRH antagonist (CA), and control group with PPOS (CB), respectively (**Figure 1**). 82 patients who had received an FET (total of 180 FETs) were evaluated for pregnancy outcomes. **Supplementary Table 1** shows detailed information for all patients and subgroups of patients.

### Patient Characteristics

**Table 1** shows patient characteristics of overall study patients and stratified by stimulation protocols, patients in the study group (mild stimulation with combination of CC and letrozole) were older (42.62 ± 3.03 vs. 40.72 ± 3.3 years, *p*<0.0001), and had a lower AMH level (0.38 ± 0.3 vs. 0.58 ± 0.32 ng/mL, *p*<0.0001) and a higher FSH level at baseline (13.43 ± 8.68 vs. 10.26 ± 4.49 pg/mL, *p*=0.001) than those of the control group (COS).

**Table 2** presents patient characteristics among the patients having embryo transfer cycles (n=82). Among these patients, the study group patients were older (42.71 ± 3.36 vs. 40.66 ± 3.11 years, *p*<0.0001), and had a lower AMH level and thinner endometrial thickness at the date of FET (8.64 ± 1.58 vs. 9.87 ± 2.05, *p*<0.0001) compared to those in the control group.

### Cycle Stimulation, Cancellation, and Ovarian Stimulation Outcomes

As shown in **Table 1**, compared to the control group, the study group had a lower utilization of gonadotropins (668.92 vs. 1,555.71, *p*<0.0001; in stimulation cycles without corifollitropin alfa [Elonva^®^]), longer duration for ovarian stimulation (10.27 ± 2.98 vs. 9.00 ± 2.19 days, *p*=0.0005), higher cancellation rate (12.38% vs. 5.61%, *p*<0.0001), and lower E2 level at the day of hCG administration (250.85 ± 257.81 vs. 645.49 ± 392.35 pg/mL, *p*=<0.0001). The mean duration of CC and letrozole co-administration in the study group was 9.31 ± 2.68 days.

In terms of stimulation outcomes, compared to the control group, the study group had lower numbers of pre-ovulatory follicles (2.00 ± 1.23 vs. 2.37 ± 1.23, *p*=0.0066) and oocytes retrieved (1.83 ± 1.17 vs. 2.37 ± 1.23, *p*=0.0017), and lower rates of FORT (58.6% vs. 68.38%, *p*=0.0093) and MOOR (44.29% vs. 52.88%, *p*=0.0386) but achieved a higher top-quality MII oocyte ratio (66.7% vs. 54.59%, *p*=0.0444) and a similar fertilization rate (91.67% vs. 89.04%, *p*=0.4660).

With adjustment for significant differences in baseline characteristics between groups (e.g., maternal age, AMH, basal FSH, length of ovarian stimulations, E2 level; shown in **Table 1**), there is no statistical difference in achieving a pre-ovulatory follicle, a MII oocyte, a top-quality MII oocyte, and a 2PN embryo between the study and control groups and between the protocol A and B groups (**Table 3**).

**Table 3:** Results of embryo outcomes between treatment protocol groups obtained using multivariable generalized equation model analyses with adjusted repeated stimulation cycles for a given patient.

Comparison	Achievement of pre-ovulatory follicle			Achievement of MII oocyte			Achievement of top-quality MII oocyte			Achievement of embryo with 2PN		
	n^1^	OR (95% CI)	*p*-value	n^2^	OR (95% CI)	*p*-value	n^3^	OR (95% CI)	*p*-value	n^3^	OR (95% CI)	*p*-value
Group comparison												
Study group versus control group (ref.)	726	1.27 (0.89, 1.82)	0.1803	692	0.90 (0.61, 1.33)	0.6074	345	1.27 (0.79, 2.03)	0.3262	345	1.64 (0.73, 3.69)	0.2316
Protocol A versus protocol B (ref.)	726	1.01 (0.77, 1.32)	0.9624	692	1.00 (0.74, 1.34)	0.9761	345	0.67 (0.44, 1.01)	0.0567	345	0.51 (0.23, 1.10)	0.0855
Subgroup comparison (ref.: study group with protocol B)												
Control group with protocol A vs. ref.	509	0.83 (0.57, 1.21)	0.3247	488	1.10 (0.71, 1.70)	0.6804	238	0.65 (0.38, 1.10)	0.1097	238	0.39 (0.13, 1.20)	0.1012
Control group with protocol B vs. ref.	344	0.72 (0.45, 1.18)	0.1912	320	1.28 (0.78, 2.08)	0.3258	154	1.09 (0.62, 1.93)	0.7631	154	0.57 (0.16, 2.04)	0.3890
Study group with protocol A vs. ref.	347	1.09 (0.7, 1.69)	0.7067	316	1.08 (0.69, 1.70)	0.7407	141	0.83 (0.37, 1.85)	0.6433	141	0.43 (0.11, 1.76)	0.2396

MII, metaphase II mature oocyte retrieved; 2PN, two-pronuclear zygote; OR, odds ratio; CI, confidence interval; study group, patients received cotreatment of clomiphene citrate and letrozole in mild ovarian stimulation; control group, patients received conventional ovarian stimulation; protocol A, patients received ovarian stimulation with gonadotropin-releasing hormone GnRH antagonist protocol; protocol B, patients received ovarian stimulation with progestin-primed ovarian stimulation (PPOS) protocol.

•n^1^ refers to total number of follicles. n^2^ refers to total number of oocytes. n^3^ refers to total number of mature embryos.

The study group is more likely to have canceled stimulation cycles compared to the control group (OR: 4.65, 95% CI: 1.14-19.03, *p*=0.0327). There is no difference in the cancellation of stimulation between the protocol A and B groups and between the study group receiving PPOS and patients receiving other types of treatment (**Supplementary Table 2**).

### Pregnancy Outcomes

**Table 2** indicates no difference in crude pregnancy rates (i.e., clinical and ongoing pregnancies) between the study and control groups and between the protocol A and B groups. **Table 4** shows that after adjustment for significant differences in baseline characteristics between groups (e.g., maternal age, AMH; shown in **Table 2**), the study group versus the control group had an insignificant increase in successful pregnancy (ORs [95% CIs]: 1.13 [0.55, 2.32] and 1.50 [0.65, 3.49] for clinical and ongoing pregnancies, respectively), the protocol A group had insignificantly lower successful pregnancies compared to the protocol B group, and the study group receiving PPOS had insignificantly lower successful pregnancies than those for the other protocols.

**Table 4:** Results of IVF pregnancy outcomes between treatment protocol groups obtained using generalized equation model analyses with adjusted repeated embryo transfer cycles for a given patient.

Comparison	Ongoing pregnancy		Live birth	
	OR (95% CI)	*p*-value	OR (95% CI)	*p*-value
Group comparison				
Study group versus control group (ref.) (180 FET cycles)	1.13 (0.55, 2.32)	0.7455	1.50 (0.65, 3.49)	0.3429
Protocol A versus protocol B (ref.) (180 FET cycles)	0.77 (0.37, 1.58)	0.4719	0.66 (0.29, 1.54)	0.3365
Subgroup comparison (ref.: study group with protocol B)				
Control group with protocol A vs. ref. (150 FET cycles)	0.71 (0.29, 1.76)	0.4590	0.5 (0.17, 1.43)	0.1949
Control group with protocol B vs. ref. (102 FET cycles)	0.74 (0.25, 2.20)	0.5892	0.47 (0.14, 1.58)	0.2221
Study group with protocol A vs. ref. (106 FET cycles)	0.61 (0.20, 1.83)	0.3770	0.47 (0.13, 1.66)	0.2390

OR, odds ratio; CI, confidence interval; FET, frozen embryo transfer; study group, patients received cotreatment of clomiphene citrate and letrozole in mild ovarian stimulation; control group, patients received conventional ovarian stimulation; protocol A, patients received ovarian stimulation with gonadotropin-releasing hormone GnRH antagonist protocol; protocol B, patients received ovarian stimulation with progestin-primed ovarian stimulation (PPOS) protocol.

## Discussion

This study provides the first evidence on clinically important outcomes (i.e., numbers and quality of follicles, oocytes, and embryos, and pregnancy) for mild stimulation with the co-administration of CC and letrozole among POSEIDON Group 4 patients receiving IVF treatment. The results show that despite the higher age and lower baseline AMH level of patients receiving mild stimulation with the co-administration of CC and letrozole (study group), the top-quality MII oocyte ratio was higher among these patients and the fertilization rate and successful pregnancy were not inferior compared to those for patients receiving COS (control group). The usage of gonadotropins in the study group was significantly reduced compared to that in the control group. These findings support the role of co-administration of CC and letrozole in the mild stimulation for poorest ovarian responders undergoing IVF treatment and highlight the potential benefit of this regimen owing to its lower cost (i.e., reduced dosages of gonadotropins, low acquisition costs of CC and letrozole) as well as better tolerance with oral administration (i.e., CC and letrozole) versus injectable conventional regimens.

### Pharmacological Perspective on Synergistic Effect of Co-Administration of CC and Letrozole in Mild Stimulation Protocol

The favorable effects of the co-administration of CC and letrozole in the context of treating POR patients can be explained by several mechanisms. CC, a nonsteroidal selective estrogen receptor modulator, decreases negative estrogen feedback to trigger normal hypothalamus compensatory mechanisms, alter the pattern of GnRH secretion, and facilitate endogenous FSH release, all of which in turn stimulate ovarian follicular growth and facilitate the early antral follicle transition (19, 20). As an alternative to CC, letrozole was introduced in assisted reproductive technology for ovulation induction. Owing to aromatase inhibitory effects (e.g., decrease of hypothalamus estrogen positive feedback, increase of ovarian androgen concentration), letrozole induces multiple follicle ovulation and improves oocyte quality. The increase of intraovarian androgen by letrozole is particularly important to prevent follicular atresia during the preantral-early antral transition phase, and stimulate granulosa cell mitosis and proliferation, which therefore facilitates follicle growth with improves oocyte quality (21). The combination of CC and letrozole is thus expected to provide synergistic effects for folliculogenesis and strengthening new waves of follicular recruitment, enhancing the overall ovarian response (22).

### Comparison With Previous Studies on Mild Ovarian Stimulation Using CC or Letrozole

Several randomized clinical trials have examined the effect of mild ovarian stimulation with CC or letrozole among POR patients undergoing IVF treatment (5, 7, 8, 23, 24).The largest trial, which included 695 POR patients who were randomized into a mild stimulation protocol with CC 100 mg/daily on days 2-6 of stimulation, 150 IU/daily gonadotropins from day 5, and a GnRH-antagonist or COS, showed lower numbers of oocytes retrieved and MII oocytes but a significantly higher cancellation rate for the mild stimulation group versus the COS group (5). Another trial of 95 POR patients (defined by Bologna criteria) randomized into three groups, namely 450 IU gonadotropins/daily GnRH antagonist protocol, 300 IU gonadotropins/daily GnRH antagonist protocol or 150 IU gonadotropins/daily + 5mg/daily letrozole GnRH-antagonist protocol on days 3-7 of ovarian stimulation, revealed no difference in the number of oocytes retrieved and clinical pregnancy rate across the three groups (8). Considering these results, the consensus statement in the 2018 American Society for Reproductive Medicine guidelines for poor ovarian responders receiving IVF treatment suggests no difference in clinical pregnancy outcomes between mild ovarian stimulation using low-dose gonadotropins with an oral superovulation agent (either CC 100 mg/daily on days 2-6 or letrozole 2.5 mg/daily on days 3-7 of the stimulation cycle) and conventional gonadotropin protocols. The 2020 European Society of Human Reproduction and Embryology guideline on Ovarian Stimulation for IVF/ICSI in mild stimulation suggests that CC in combination with gonadotrophins or gonadotrophins stimulation alone is comparable to COS based on evidence that showed similar numbers of oocytes retrieved and live birth rates between mild stimulation with CC and conventional protocols (25–27). However, the ESHRE does not recommend the use of letrozole in mild stimulation protocols due to the lack of improvement of clinical outcomes with ovarian stimulation (25) and safety concerns regarding letrozole (i.e., possible teratogenicity). But, more recently, a systematic review and meta-analysis supports the use of letrozole in fertility treatment (including its adjuvant role to gonadotropins in ovarian stimulation among women receiving IVF treatment) because there is no evidence for increased congenital malformation and pregnancy loss risks with letrozole compared to CC or other fertility drugs and natural conceptions (28). For ovulation induction, letrozole may thus be considered as a first-line agent given its therapeutic benefits and no evidence of harm to the fetus (28).

In the present study, CC and letrozole were co-administrated in mild ovarian stimulation, which yielded non-inferiority in the clinical outcomes of the stimulation and pregnancy outcomes following ETs and resulted in no clinically significant adverse events observed. Additionally, consistent with previous studies (5, 6) using a single super-ovulating agent (i.e., CC or letrozole), the cancellation of mild stimulation with combination of CC and letrozole was significantly higher than that for conventional regimens. This may be explained by the more advanced maternal age (42.62 vs. 40.72 years, *p*<0.0001) and lower baseline AMH levels (0.38 vs. 0.58 ng/mL, *p*<0.0001) in the mild stimulation group compared to the conventional group.

Moreover, given potential differences between the PPOS and GnRH antagonist protocols (11–14), the present study further stratified patients by these two protocols. We found that the effects of the co-administration of CC and letrozole were not modified by these two protocols. That is, patients with the co-administration of CC and letrozole, regardless of protocol (PPOS or GnRH antagonist), had a better top-quality MII oocyte ratio and insignificantly better pregnancy outcomes (**Table 4**).

### Study Limitations

This study used a retrospective design and thus may be affected by unavoidable biases. However, we carefully selected patients who are appropriate for the study aims according to inclusion and exclusion criteria, stratified them by treatment protocol for comparison, applied advanced GEE model analyses to account for potential dependency across repeated IVF cycles (i.e., stimulation cycles, ET cycles) for a given patient, and adjusted for significant confounders for study outcomes using multivariable GEE models. These efforts ensure the validity of our findings. Study participants were all from a medical center and may thus be representative of a subset of patients with severe forms of infertility problems and possible experience with various fertile treatments. As a result, selection bias may not be eliminated. However, this study focused on POR patients, who have severe infertility and are likely to end up in advanced medical institutions (e.g., our fertility center) for treatment. The patients from our fertility center might thus be representative of POR cases in southern Taiwan. And, since not all study patients who achieved a successful pregnancy received prenatal exams and gave birth in the study hospital, we were not able to provide detailed patient’s follow-up data on their newborns including body weight and metabolic parameters, or to study potentially long-term teratogenic events of treatment. The safe profile associated with the CC and letrozole treatment on maternal and neonatal outcomes is thus warranted for future research. Furthermore, the present study did not estimate the average number of cycles required to obtain a pregnancy in POSEIDON Group 4 patients and current evidence on this estimate is also lacking, which highlights a need for future research. Lastly, due to the limited number of POR cases in clinical practice, our study sample sizes for individual treatment protocols were low. Future prospective randomized trials with large patient populations are needed to corroborate the findings of this study.

## Conclusion

This study provides preliminary evidence on the effect of cotreatment with CC and letrozole in mild stimulation for POSIDONE Group 4, a group of infertile patients with poor prognosis. Although the benefit of this combination is not marked, the effect is clinically measurable, leading to a higher top-quality MII oocyte ratio, comparable fertilization rate, and potentially an increased rate of pregnancy compared to those obtained with COS regimens. These promising results highlight the need for future large prospective trials and cost-effectiveness studies to confirm the clinical efficacy and value of cotreatment with CC and letrozole in mild stimulation for POSIDONE Group 4 patients undergoing IVF treatment.

## Data Availability Statement

The datasets presented in this article are not readily available because it contained individual patient, identifiable data. Requests to access the datasets should be directed to the first authors: Hsin-Ta Lin (linshingda@gmail.com) and Meng-Hsing Wu (mhwu68@mail.ncku.edu.tw).

## Author Contributions

All authors listed have made a substantial, direct, and intellectual contribution to the work and approved it for publication.

## Funding

This work was supported by the Ministry of Science and Technology, Taiwan, under grant MOST 109-2320-B-006-047-MY3 (recipient: Huang-tz Ou). The sponsor had no role in the design and conduct of the study; collection, management, analysis, and interpretation of the data; preparation, review, or approval of the manuscript; and decision to submit the manuscript for publication.

## Conflict of Interest

The authors declare that the research was conducted in the absence of any commercial or financial relationships that could be construed as a potential conflict of interest.

## Publisher’s Note

All claims expressed in this article are solely those of the authors and do not necessarily represent those of their affiliated organizations, or those of the publisher, the editors and the reviewers. Any product that may be evaluated in this article, or claim that may be made by its manufacturer, is not guaranteed or endorsed by the publisher.

## Supplementary Material

The Supplementary Material for this article can be found online at Figshare. Supplementary Figure 1 (doi: 10.6084/m9.figshare.14626053.v5); Supplementary Table 1 (10.6084/m9.figshare.14626407.v3); Supplementary Table 2 (10.6084/m9.figshare.14626425.v4).
